# Research Progress on Slit/Robo Pathway in Pancreatic Cancer: Emerging and Promising

**DOI:** 10.1155/2020/2845906

**Published:** 2020-06-29

**Authors:** Cheng Ding, Yatong Li, Cheng Xing, Hanyu Zhang, Shunda Wang, Menghua Dai

**Affiliations:** ^1^Department of General Surgery, Peking Union Medical College Hospital (PUMCH), Peking Union Medical College & Chinese Academy of Medical Sciences, Beijing 100730, China; ^2^National Translational Medicine of China, Beijing 100730, China

## Abstract

Pancreatic cancer is a highly malignant digestive system tumor which is the leading cause of cancer-related deaths. The basic and clinical research of pancreatic cancer has made great progress in recent years, and kinds of signaling pathways have been found in the tumorigenesis and progression in pancreatic cancer. The Slit glycoprotein (Slit) and Roundabout receptor (Robo) signaling pathway acts as a neural targeting factor with the axonal remnant, axon guidance, and inhibition of neuronal migration in the nervous system. In recent years, it has been found that the Slit/Robo signaling pathway has different degrees of expression changes in various tumor cells. In different tumor cells, the signaling pathway gene expression is different and regulates tumor angiogenesis, cell invasion, metastasis, and nerve infiltration. Herein, we summarize the mechanisms of the Slit/Robo pathway in the development and progression of pancreatic cancer, in order to have more understanding of the role of Slit/Robo in pancreatic cancer.

## 1. Introduction

Pancreatic ductal adenocarcinoma (PDAC), generally known as pancreatic cancer, is a highly lethal malignancy disease. According to the American Cancer Society, pancreatic cancer ranks fourth in cancer-related deaths and is expected to become the second most common malignant tumor in mortality in 2030 [1, 2]. In the United States, pancreatic cancer is expected to kill 23,800 men and 21,950 women each year [1–3]. The newest statistics showed that the overall 5-year survival rate of pancreatic cancer was about 9% [1]. Pancreatic cancer is characterized as aggressive local invasion, early lymphatic and hematogenous dissemination, distant metastasis, and chemotherapeutic resistance. The difficulty of early diagnosis is a major obstacle to long-term survival. Nearly 80% of patients have no opportunity of surgery at the time of diagnosis. Despite extensive research in adjuvant therapy in recent decades, there has been no breakthrough in overall treatment outcomes due to the dense interstitial and chemotherapy resistance of pancreatic cancer. The high invasiveness of pancreatic cancer enables the rapid tumor to grow rapidly and metastasis to the liver, peritoneum, and lungs. Indeed, metastasis is the main cause of patient death. Several genetic and environmental risk factors play roles in pancreatic tumorigenesis, including age, gender, ethnicity, smoking status, alcohol abuse, obesity, diet, diabetes, and chronic pancreatitis. Pancreatic cancer is the result of complex interactions between multiple genetic mutations and these environmental factors [4–6].

Pancreatic cancer results from the complex interaction of multiple genetic mutations and environmental factors. A variety of oncogenic signaling pathways are known to be involved in pancreatic carcinogenesis including the WNT/*β*-catenin, Hedgehog, nuclear factor-*κ*B (NF-*κ*B), hepatocyte growth factor (HGF), and transforming growth factor (TGF-*β*) pathways [7]. Enhanced expression and signaling via these pathways lead to increased proliferation, invasion, metastasis, and immune evasion by pancreatic cancer cells.

In recent years, the Slit glycoprotein and Roundabout (Robo) signaling pathway, which was originally identified through its role in axon guidance, has been found to contribute to the development and progression of many tumors. As such, the Slit/Robo pathway has become an area of intense focus in cancer research, and inhibition of this pathway has been shown to have beneficial effects against various tumors [8–11]. About 5%–15% of pancreatic cancer patients harbor mutations in Slit and/or Robo genes, and about 48% of patients exhibit altered Slit or Robo gene methylation, suggesting that aberrations in this signaling pathway may be a common characteristic of pancreatic cancer [12, 13]. Upregulation of WNT/*β*-catenin signaling, a conserved pathway that plays a role in cell fate decisions, has also been implicated in pancreatic cancer, and recent data suggest that the Slit/Robo pathway may regulate WNT/*β*-catenin-dependent signaling [12].

An in-depth analysis of these signaling pathways could not only increase our understanding of the molecular events underlying pancreatic cancer but also potentially identify novel therapeutic targets. In this brief review, we summarize our current understanding of the role and related mechanisms of the Slit/Robo pathway in pancreatic cancer.

## 2. The Components of Slit/Robo Proteins

The Robo gene, which encodes a transmembrane receptor, was first found in *Drosophila*. And then, the Slit protein was identified as the ligand. In 1984, Nüsslein-Volhard et al. [14] first discovered the presence of the axon-directing molecule Slit gene in *Drosophila*. Larvae and his colleagues first cloned it successfully in the *Drosophila* nervous system in 1988 [15]. The Slit gene family has been found in a variety of animals including nematodes, mice, zebrafish, and humans [16–19]. Roundabout was discovered by Kidd et al. in 1998 and confirmed to be a receptor for Slit, which combine to exert axonal targeting rejection [20]. There is only one Slit gene in invertebrates, and there are three Slit genes in vertebrates, namely, Slit1, Slit2, and Slit3. The human Slit1 gene is located on chromosome 10q24.1, the Slit2 gene is located at 4p15.31, and the Slit3 gene is at 5q34∼q35.1. The Slit gene is highly conserved among different species. The protein sequence homology of the nematode Slit and *Drosophila* Slit is 41%. The sequence homology of the three Slits of vertebrates with the Slit of *Drosophila* and *C. elegans* is 41%∼44%, and 39%∼41%.

The Slit gene encodes a secretory glycoprotein with a relative molecular weight of approximately 200 kDa. Slit1 expression is restricted to neural tissue, while Slit2 and Slit3 are more widely expressed in tissues such as kidney, lung, and skin other than the nervous system. The Slit protein consists of four distinct domains at the N-terminus (D1–D4) with leucine-rich repeats (LRR), six EGF-like sequences (EGF), a laminin-G domain, and a C terminal with a cysteine-rich knot. Proteolytic enzymes cleave Slit at the EGF-like domain, releasing an active N-terminal fragment [21–23].

The Robo receptor family is a single pass, a transmembrane protein, belonging to the immunoglobulin superfamily. There are four major members in mammals: Robol, Robo2, Robo3, and Robo4 [24, 25]. Robo1, Robo2, and Robo3 are similar in structure, including three parts: the extracellular domain which contains five immunoglobulin- (Ig-) like domains followed by three fibronectin domains (Fn III), transmembrane regions, and the intracellular region which consists of four conserved cytoplasmic (CC) domains. The structure of Robo4 is quite special. There are only two Ig-like domains and two Fn III in the extracellular region, and the intracellular region consists only of motifs CC0 and CC2. Robo1– Robo3 are expressed in many tissues during development and particularly in the nervous system, while Robo4 is particularly expressed in endothelial cells.

## 3. The Physiological Function of the Slit/Robo Pathway

Nerves and blood vessels are physically and physiologically connected, and the arteriovenous network and neural systems are interweaved in most organisms. In the developing nervous system, neuronal axons sense environmental cues through the growth cone and form pseudopod structures attached to the filament at the front of the growth cone through receptor/ligand interactions. Slit/Robo signaling plays an important role in axonal guidance by acting as a repulsive cue, preventing axonal migration to inappropriate locations during the formation of the nervous system [15].

Robo4 is exclusively expressed by endothelial cells. Dickinson et al. [21] found that Robo4 mRNA is expressed on placental arterioles and venules, Robo4 protein is expressed in trophoblast cells during angiogenesis, and Slit3 mRNA is also detected in placental tissues. There is also evidence that the Slit2 protein localizes in the syncytiotrophoblast of placental villi, while Robo1 is expressed in the syncytiotrophoblasts of the placental villi and the trophoblast capillaries. This suggests that Slit2 may be secreted by trophoblast cells in a paracrine manner and regulate endothelial cell function and angiogenesis by binding to Robo1 on endothelial cells. In the oxygen-induced mouse retinopathy model, the Robo4 gene expression level was significantly higher than that of the control group, while the Slit1-3 and Robo1-3 gene expression level was not significantly different from the control group. Further studies have shown that recombinant Slit2 can reduce choroidal angiogenesis and retinal angiogenesis, and knocking out Robo4 will alter these effects [26].

The formation of tumor angiogenesis has a great role in promoting the occurrence, development, and metastasis of tumors. It has been found that there are two receptors, Robo1 and Robo4 [27–29], in vascular endothelial cells. In the Slit/Robo signaling pathway, they have different effects on vascular endothelial cells, which in turn affects the formation of new blood vessels. In promoting tumor angiogenesis, Wang et al. [30] found that human umbilical vein endothelial cells (HUVECs) have Robo1 receptor, and Slit2 secreted by tumor cells can bind to it, attracting HUVCE to migrate to the tumor and induce tumor angiogenesis. This process relies on the involvement of Robo1 and phosphatidylinositol kinase. If the activity of Robo1 is blocked it will reduce the microvascular formation and reduce the tumor volume of human malignant melanoma A375 cells [30].

Seth et al. [31] found that Robo4 expression was detected in vascular endothelial cells of melanoma, kidney cancer, lung cancer, liver cancer, and other malignant tumors. The authors also found that Robo4 can inhibit vascular endothelial growth factor and fibroblast growth factor-induced cell migration by activating Slit2. Stella et al. [32] reported that the Slit/Robo1 signaling pathway is able to inhibit motility-induced endothelial cell migration. Hepatocyte growth factor- (HGF-) induced cell migration, invasion, and angiogenesis can be promoted by inactivating the Slit2 gene by siRNA or aberrantly expressing the Robo gene.

In addition to these functions, the Slit/Robo signaling pathway regulates a number of other processes involved in cell growth, including myogenesis, kidney induction, leukocyte migration, cardiac tube formation, and vascular injury and repair [33–35].

## 4. Slit/Robo Pathway and the Development of Pancreas

Escot et al. [36] showed that Robo1 and Robo2 play important roles in the development of the pancreas. Simultaneous knockout of Robo1 and Robo2 resulted in a significant reduction in pancreatic volume in the mouse, especially in the head of the pancreas, in the developing embryo; however, knocking out Robo1 or Robo2 alone will not have this effect. And in mice knocked out of Robo1/2, some pancreatic progenitor cells acquired the characteristics of liver progenitor cells. Further genetic lineage tracking revealed that some of the Robo1/2 knockout pancreatic cells migrated during the embryonic stage and formed part of the liver. The authors' research indicates that Robo receptors play an important role in maintaining pancreatic tissue identity. In further in vivo and in vitro studies, Escot et al. showed that Robo inactivation led to a metastable state of endoderm cells and affected the proliferation of pancreatic progenitor cells via interaction with the YAP/TEAD pathway.

## 5. Slit/Robo Pathway and Pancreatic Carcinoma

In a study of 142 patients with early (clinical stage I/II) pancreatic cancer who did not receive neoadjuvant therapy, Biankin et al. [12] found that mutations in components of the Slit/Robo pathway are not only relatively common but also closely related to patient prognosis. In these patients, longer overall survival was observed in patients with high tumor expression of Robo2 compared with patients with low Robo2 expression, whereas the opposite pattern was observed for Robo3 expression [37].

And then, several scholars further explored the role of the Slit/Robo pathway in pancreatic cancer. Nones et al. [13] found that the Slit/Robo pathway has methylation changes in 48% of pancreatic cancer patients by genome-wide sequencing of pancreatic cancer specimens. Gohrig et al. [38] found that Slit2 mRNA is downregulated in pancreatic cancer and is associated with lymph node metastasis. Further experiments have shown that, in MiaPaCaTR-Slit2, Slit2 can inhibit direct invasion and metastasis of tumor cells. In the mouse model of pancreatic cancer, high expression of Slit2 can inhibit the growth of pancreatic tumors, reduce its volume, inhibit tumor invasion of the stomach and duodenum, and inhibit lymphatic metastasis, distant metastasis, and tumor angiogenesis. Knocking out Robo1 can enhance the invasion and metastasis of pancreatic tumors [38]. Meanwhile, the authors also confirmed that the expression of Slit2 can inhibit the invasion ability of pancreatic tumors to peripheral nerves [38].

Sabatier et al. [37] showed that Robo3 is an inhibitor of Robo2 and that high expression of Robo3 mRNA is associated with poor prognosis in patients with pancreatic cancer. Subsequently, Han et al. [39] found that Robo3 protein expression was elevated in pancreatic cancer compared with normal pancreatic tissue. Moreover, its expression increased in parallel with the tumor stage, and Robo3 levels correlated negatively with those of Robo1 and Robo2. Upregulation of Robo3 expression in the human pancreatic cancer cell lines PANC-1 and Capan-1 significantly promoted their growth and invasion in mice. In the mouse model, the high expression of Robo3 promoted both tumor growth and liver metastasis.

Overall, these studies have demonstrated potentially contradictory roles for Slit/Robo signaling in pancreatic cancer (summarized in Table 1). Further studies will be necessary to clarify how this complex signaling pathway is involved in the development and progression of pancreatic cancer and the relationship to patient outcomes.

## 6. The Mechanism of Slit/Robo Signaling Pathway in Pancreatic Cancer

A variety of cell signaling pathways play well-known roles in pancreatic cancer, including WNT/*β*-catenin, TGF-*β*, NF-*κ*B, and HGF signaling pathways. Each of these plays different roles in pancreatic cancer through effects on tumor growth, migration, invasion, and metastasis.

A large number of genes regulating cell proliferation, differentiation, and tumorigenesis are transcriptionally regulated by the WNT/*β*-catenin pathway. Abnormal changes in WNT/*β*-catenin signaling have been demonstrated in various tumors such as pancreatic, cervical, lung, gastric, and colorectal cancers [42–46]. High expression of Slit2 is associated with better clinical outcome in patients with pancreatic, breast, lung, and colorectal cancers, among others [38, 47–49]. One possible explanation for this observation inhibition of the WNT/*β*-catenin pathway by Slit2/Robo signaling, which enhances the formation of *β*-catenin and E-cadherin complexes, increases tumor cell adhesion and inhibits tumor invasion and migration, thereby improving patient prognosis. Inhibition of the WNT/*β*-catenin pathway may thus be the main mechanism by which the Slit/Robo pathway inhibits pancreatic cancer growth. Robo3, the inhibitor of Robo2, activates the WNT/*β*-catenin pathway, thus promoting pancreatic cancer growth and invasion [39].

HGF is a 90-kDa glycoprotein secreted by mesenchymal cells [50]. The HGF transmembrane cell surface receptor, MET, is a product of the proto-oncogene c-MET and is usually expressed on epithelial cells. Binding of HGF to MET activates several signaling pathways, including MAPK and PI3K, which regulate the proliferation, invasion, and migration of cancer cells, including pancreatic cancer [51]. In mouse models of pancreatic cancer, HGF inhibitors reduce tumor growth, invasion, and distant metastasis. Slit/Robo signaling can downregulate MET signaling, thereby countering the effects of HGF–MET interactions on the tumor cells.

Pancreatic cancer is composed of highly heterogeneous tissue with a large interstitium. The tumor stroma is mainly composed of pancreatic stellate cells, myofibroblasts, nerve fibers, hyaluronic acid, fat cells, immune cells, inflammatory cells, and other components. The interstitial component occupies more than half of the tumor volume, and the cancer cells are scattered between the interstitial components with immature adenoid structures. The tumor microvessels are highly compressed, making it difficult for chemotherapeutic drugs to reach the tumor cells at efficacious concentrations. As a result, most drug treatments have poor efficacy.

**Figure 1:**
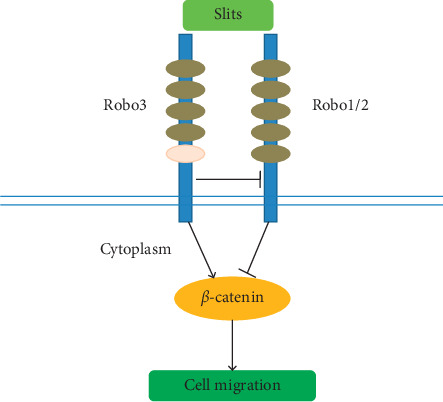
Structure of the Slit/Robo protein. Robos are receptors of Slits proteins. Slit/Robo1/2 suppresses the activity of WNT/*β*-catenin, while the Robo3 is an inhibitor of Robo2.

**Figure 2:**
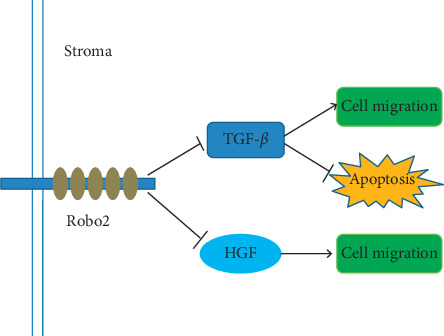
The schematic representation of the Slit/Robo signaling pathway in pancreatic cancer. Robo2 suppresses the stroma of pancreatic cancer, including TGF-*β* and HGF.

TGF-*β* plays a role in promoting the progression, migration, invasion, and metastasis of pancreatic cancer [52]. At present, TGF-*β* inhibitor combined with gemcitabine chemotherapy has been used in the clinical trial of pancreatic cancer treatment and has achieved promising outcomes. Gemcitabine combined with TGF-*β* inhibitors significantly prolonged overall survival and disease-free survival [53]. Pinho et al. [41] showed that Robo2 is expressed in the epithelium and stroma of normal adult mouse pancreas, whereas Robo2 was absent from epithelial cells in a cerulein-induced model of acute pancreatitis in the mouse. In the mouse model, TGF-*β* administration resulted in activation of the pancreatic stroma and promotion of a strong anti-inflammatory response, and this was reversed by treatment with the TGF-*β* inhibitor galunisertib. The authors of this study, therefore, defined Robo2 as a stroma suppressor gene. Figures 1 and 2 illustrate the potential involvement of Slit/Robo signaling in pancreatic cancer.

Insensitivity to chemotherapy is a crucial factor in the poor prognosis of patients with pancreatic cancer. Gemcitabine, which is often used for the treatment of pancreatic cancer [54], is a nucleotide analog and inhibits ribonucleotide reductase, leading to DNA asymmetry and inhibition of tumor cell growth. However, pancreatic cancer is highly resistant to gemcitabine, limiting its clinical utility. Studies on the relationship between the expression and function of microRNAs (miRNAs) and tumor chemosensitivity have received increasing attention in recent years [55–58]. He et al. [40] have shown that Robo1 expression is negatively correlated with microRNA-218 levels in pancreatic cancer. High expression of MicroRNA-218 can inhibit the invasion and metastasis of pancreatic cancer. Liu et al. [59] found that microRNA-218 can enhance the sensitivity of pancreatic cancer cells to gemcitabine treatment and promote tumor cell apoptosis. Low Robo1 expression levels may thus contribute to gemcitabine resistance in pancreatic cancer.

## 7. Conclusion

Despite extensive basic and clinical research, pancreatic cancer remains a malignancy with a very poor prognosis. The Slit/Robo signaling pathway, originally described as an axon-directing factor, has been shown in recent years to play additional roles in cell growth, migration, and survival. Slit/Robo expression is often downregulated or undetectable in advanced stage cancers, and Slit/Robo signaling is considered to act as a tumor suppressor to inhibit tumor invasion and metastasis. However, the role of Slit/Robo signaling in pancreatic cancer is currently controversial and remains to be clarified. The outstanding questions to be addressed include which specific Slit and Robo family proteins play the leading role(s) in pancreatic cancer and which additional pathways, such as cytokine-and growth factor-activated pathways, modulate signaling *via* Slit/Robo to affect tumor cell invasion, metastasis, and angiogenesis. Given that the Slit/Robo pathway regulates a variety of oncogenic signaling events, targeting of this pathway could have potential as a novel anticancer therapy.

## Figures and Tables

**Table 1:** Summary of Slit/Robo pathway expression and function in PDAC.

Author	Gene	Expression	Function	Metastasis
Gohrig et al. [38]	Slit2	Downregulation	Tumor and invasion suppressor	Suppress
He et al. [40]	Robo1	Upregulation	Promote the lymphatic metastasis of pancreatic cancer	Promote
Pinho et al. [41]	Robo1	Upregulation	Corresponding to poor prognosis	—
Pinho et al. [41]	Robo2	Downregulation	Stroma suppressor gene	Suppress
Biankin et al. [12]	Robo2	Downregulation	Tumor suppressor and high expression is associated with better prognosis	Suppress
Biankin et al. [12]	Robo3	Upregulation	Associated with poor prognosis	Promote
Han et al. [39]	Robo2	Downregulated with tumor stage	—	—
Han et al. [39]	Robo3	Upregulated with tumor stage	Promote growth and metastasis of pancreatic cancer	Promote
